# Chromium Contamination and Health Risk Assessment of Soil and Agricultural Products in a Rural Area in Southern China

**DOI:** 10.3390/toxics11010027

**Published:** 2022-12-27

**Authors:** Shun’an Xu, Chao Yu, Qiong Wang, Jiayuan Liao, Chanjuan Liu, Lukuan Huang, Qizhen Liu, Zheyu Wen, Ying Feng

**Affiliations:** 1 MOE Key Laboratory of Environment Remediation and Ecological Health, College of Environmental and Resource Sciences, Zhejiang University, Hangzhou 310058, China; 22014150@zju.edu.cn (S.X.); wq122506@zju.edu.cn (Q.W.); 22214125@zju.edu.cn (J.L.); 22114161@zju.edu.cn (C.L.); 12114025@zju.edu.cn (L.H.); 12214012@zju.edu.cn (Q.L.); wzywenzheyu@zju.edu.cn (Z.W.); 2 Livestock Industrial Development Center of Shengzhou, Shaoxing 312400, China; 33406041@qq.com; 3 College of Ecology, Taiyuan University of Technology, Taiyuan 030024, China

**Keywords:** Cr, rice, legume, vegetable, health risk assessment, farmland

## Abstract

With the rapid development of industry, chromium (Cr) pollutants accumulate constantly in the soil, causing severe soil Cr pollution problems. Farmland Cr pollution hurts the safety of agricultural production and indirectly affects human health and safety. However, the current situation of Cr pollution in farmland soil and crops has not been detailed enough. In this study, the evaluation of Cr potential risk in soil-crop systems was conducted in a rural area that was affected by industry and historic sewage irrigation. Ten different crops and rhizosphere soils were sampled from four fields. The results showed that Cr contents in farmland soil exceeded the national standard threshold in China (>21.85%), and the Cr content in edible parts of some agricultural products exceeded that too. According to the PCA and relation analysis, the Cr accumulation in edible parts showed a significant correlation with soil Cr contents and available potassium contents. Except for water spinach, the target hazard quotient (THQ) of the other crops was lower than 1.0 but the carcinogenic health risks all exceeded the limits. The carcinogenic risks (CR) of different types of crops are food crops > legume crops > leafy vegetable crops and root-tuber crops. A comprehensive assessment revealed that planting water spinach in this area had the highest potential risk of Cr pollution. This study provided a scientific and reliable approach by integrating soil environmental quality and agricultural product security, which helps evaluate the potential risk of Cr in arable land more efficiently and lays technical guidelines for local agricultural production safety.

## 1. Introduction

Chromium enters the environment through natural processes and anthropogenic activities including mining, smelting, metal processing, industrial production, and agricultural activities, resulting in the pollution and the destruction of ecosystems [1]. Among many sources of pollution, tanning is the main factor causing pollution. This process requires the use of Cr-containing compounds to tan the leather, and the utilization rate of Cr in this process is only 60–75%, so Cr-contained water will be discharged and the environment will be contaminated [2].

Besides staple food such as rice or wheat, vegetables play an important role in the Chinese daily diet as indispensable cash crops. However, various human activities, such as mining, smelting and other industrial processing, pesticides, automobile exhaust and fertilization, especially the extensive use of organic livestock and poultry manure as annual traditional agricultural fertilizer, led to an increase in the concentration of heavy metals in China [3]. Heavy metal pollution in the soil directly affected the growth quality of crops. According to the National Soil Pollution Survey Bulletin in 2014, the total over-standard rate of soil was 16.1%, and the over-standard rate of Cr in soil was 1.1% in China, among which slight Cr pollution accounted for a large proportion; the amount of soil contaminated by Cr was up to 15 million tons nationwide [4,5]. Cr pollution also varied across China. According to the investigation, Cr in Ningbo City was the most important element exceeding the standard in stem vegetables. The samples with Cr contamination accounted for 45.0% of the collected samples and the exceeding rate reached 13.2% [6].

Cr exists in the environment in a variety of valence states, with the most stable forms Cr (VI) and Cr (III) having different properties among which hexavalent Cr causes the main pollution toxicity [7,8]. The most common Cr compounds in soil are HCrO_4_ and CrO_4_^2−^, which are easily absorbed by plants and contaminate the soil [9]. As Cr is absorbed by the plant, it has negative effects on the growth and development of plant tissues. Cr enters the plant through the root system, and some are transported to the aboveground parts of the plant along with nutrients, affecting the growth of stems and leaves and other organs [10,11]. A high Cr concentration leads to root cell wilt and plasmid wall separation and induces a higher frequency of chromosomal aberrations in root tip cells, which leads to inhibited root cell division and differentiation, reducing the volume and the number of root cells [12]. In addition, the stems and leaves of plants will also show toxic effects of Cr, which can induce plant toxicity by interfering with plant growth, nutrient absorption and photosynthesis, inducing increased production of reactive oxygen species, causing lipid peroxidation and changing antioxidant activity, reducing the growth and height of branches, biomass, photosynthetic pigments and protein content in plant leaves [13,14,15].

Chromium is classified as a class A carcinogen due to its high toxicity [16]. Cr (III) is an essential nutrient trace element, non-toxic and difficult to absorb. Trivalent Cr can enhance insulin activity as receptor binding and reduce the risk of diabetes. Excessive trivalent Cr exceeding the recommended value may lead to long-term toxicity and carcinogenicity [8]. As one of the most toxic forms, Cr (VI) and its metabolites, especially chromate, enter the human body through different ways (inhalation, ingestion and skin contact), which will cause pathological changes to the human organs and systems (respiratory tract, skin, gastrointestinal tract and so on) and even increase the incidence rate and mortality of many cancers [9,17]. Prolonged human exposure can cause gastrointestinal upset, respiratory problems, kidney and liver damage, and altered genetic material, among other conditions [18]. The main pathogenesis is attributed to DNA damage, genomic instability and reactive oxygen species (ROS) production by Cr (VI). Cr (VI) induces oxidative stress and ROS production in high levels of target DNA and cell lipid content, which lead to DNA damage and lipid peroxidation, respectively [19]. Therefore, Cr pollution of farmland has a great negative effect on the safe production of crops, and it also has a serious hidden danger to human health.

In order to ensure the healthy consumption of crops and food by human beings, health risk assessments on crops and soil were conducted through various indicators developed by international organizations such as the United States Environmental Protection Agency (USEPA) and the World Health Organization (WHO) [20]. The target hazard quotient (THQ) method is a method to evaluate the effects of harmful substances on human health established by the USEPA in 2000. It is used to evaluate the health risks of single heavy metals in vegetables and leaves and the combined health risks of multiple heavy metals [21]. Chen et al. calculated and evaluated the pollution load index (PLI), potential ecological risk index (RI), and the highest target hazard quotient (THQ) of the greenhouse vegetable production system (GVP), which showed that the accumulation of cadmium (Cd) and mercury (Hg) in these GVP soils was evaluated to be more significant than other elements [22]. Estimated Daily Intake (EDI) refers to the estimated amount of a component of food consumed by the average consumer. The presence of non-carcinogenic risks is often judged in conjunction with THQ intake. Pelcová et al. assessed the health risk of Hg pollution in the edible parts of various crops to determine the accumulation rule and accumulation capacity of various crops and gave the planting guidance according to the EDI and THQ value [23]. In China, the single-factor pollution index method was used to evaluate the pollution degree of heavy metals in soil. It evaluated the pollution of a single factor in a specific area by the ratio of the actual pollution level to the standard limit value [24]. However, the study of health risks of soil-crop systems in typical Cr-polluted farmland is still rare.

Therefore, the aims of this study were to (1) compare the characteristics of Cr uptake and transportation among different crop-soil systems; (2) evaluate the health risk of different crops; so as to provide technical support for proper risk assessment and subsequent treatment of the Cr polluted farmland.

## 2. Materials and Methods

### 2.1. Sample Processing

The soil and plant samples were collected at a depth of 0–20 cm from four fields (paddy field F1, vegetable field C1, C2, C3) in a town in Zhejiang Province of China (Figure S1). The sampled farmland soils belonged to the typical subtropical monsoon climate zone, mainly consisting of paddy soil and brunisolic soil with good farming conditions which were historically sewage irrigated. It was learned from the local government that the sampled soil had a certain degree of chromium pollution, and there were a large number of industries near the sampled farmland including aquaculture, plastic products, metal forging and other industrial and agricultural industries; the sampled farmland was near the national highway. These environmental factors easily contaminate irrigation water and soil.

### 2.2. Plant Sample Analysis

Plant samples were selected with three replicates taken in each field. After cleaning up, the collected samples were weighed for the fresh weight (FW) and divided into aboveground and underground parts. The underground parts were soaked with 20 mM Na_2_EDTA for 20 min and washed three times with deionized water. Subsequently, the plant samples were killed at 10 °C for 30 min and then dried at 65 °C to constant weight. The dry weight (DW) was weighed and the water content was calculated. Then each part of the plant was ground into powder and passed through a 0.15 mm sieve for the chemical analysis.

0.1 g of plant sample (DW) was weighed and digested for 6 h at 150 °C in HNO_3_:H_2_O_2_= 5:2 (*v*/*v*) until completely clear, which was finally determined by inductively coupled plasma-mass spectrometry (ICP-MS) [25,26].

### 2.3. Soil Sample Analysis

The rhizosphere soil samples adhered to the plant roots were collected with three replicates from each site. Soil samples were ground and passed through 1.0 mm (for soil physical properties) and 0.15 mm (for soil chemical properties) sieves after natural air drying which were stored in sealed bags for the analysis of soil.

Soil pH was determined in 1:2.5 soil-water suspension determination of soil pH value [2]. Soil organic carbon (TOC) was determined by potassium dichromate oxidation and external heating method [27]. Soil total nitrogen (TN) was determined by an element analyzer. Available phosphorus (AP) in the soil was extracted by 0.5 M NaHCO_3_ (soil:solution ratio of 1:20) and determined by the molybdenum-antimony anti-colorimetric method. Available potassium (AK) was extracted by 1 M NH_4_OAC (soil:solution ratio of 1:10) with flame photometry [28]. Soil cationic exchange (CEC) was determined by spectrophotometer, using hexamminecobalt trichloride solution extraction (soil:solution ratio of 7:100) [29].

The content of total Cr in soil was determined by microwave digestion-ICP-MS according to HJ491-2019. Soil samples were digested with 7 mL of mixed acid solution (HNO_3_:HClO_4_:HF= 5:1:1 (*v*/*v*)). The microwave digestion instrument was carried out according to the fixed temperature program. After cooling, the digestion solution was diluted to be measured.

### 2.4. Evaluation Criteria

The assessment standard for heavy metal pollution in soil was conducted according to GB 15618-2018 in China, the risk screening values of Cr-contaminated soil are 250 mg·kg^−1^ in paddy fields and 150 mg·kg^−1^ in other farmlands (pH < 6.5). The risk intervention value of Cr-contaminated soil is 800 when the pH is less than 5.5 and 850 when the pH is more than 5.5 and less than 6.5, respectively. The evaluation standard for heavy metal pollution in vegetables was conducted following the National Standard of Food Safety (GB 2762-2017) of China, which provisions limits for Cr in crops. Food crops and legume crops are limited to 1.0 mg·kg^−1^; leafy vegetable crops and root-tuber crops’ quota is 0.5 mg·kg^−1^.

### 2.5. Evaluation Method for Pollution Risk

Single factor pollution index method is expressed by the following:

(1) $$P_{i}=\frac{C_{i}}{S_{i}}$$

wherein, *P_i_* is the pollution index of the calculated heavy metal elements; *C_i_* is the measured value of the heavy metal element; *S_i_* is the standard value of soil environmental quality. *P_i_* > 1, no contamination; 1 < *P_i_* ≤ 2, light pollution; 2 < *P_i_* ≤ 3, medium pollution; *P_i_* > 3, heavy pollution.

Transport factor (TF) can be used as an index to evaluate the heavy metal accumulation ability of plants [30].

(2) $$\mathrm{TF}=\frac{C_{Aboveground}}{C_{Underground}}$$

*C_Aboveground_* represents the Cr content in the leaf or stem of crops and *C_Underground_* represents the Cr content in the root of crops.

The bioconcentration factor (BCF) is an index to evaluate the capacity of plants to absorb and transfer heavy metals into the body [31].

(3) $$\mathrm{BCF}\left(\%\right)=\frac{C_{Edible\;part}}{C_{Soil}}\times 100$$

*C_Edible part_* represents the Cr content in edible parts of crops and *C_Soil_* represents the Cr content in the soil.

### 2.6. Nor-Carcinogenic Risk Assessment

The estimated dietary intake (*EDI*) of metals depends on the metal concentration of the edible part of vegetables, daily vegetable consumption, a period of time (life cycle) and body weight, which is calculated as follows:

(4) $$EDI=\frac{EF\times ED\times IR\times C}{BW\times AT}$$

*EF* represents exposure frequency (350 d·a^−1^); *ED* means exposure duration (The *ED* values for adults were 30 years, respectively) [32]; *IR* is the inhalation rate; the average daily intakes of vegetables for adult inhabitants were 0.345 kg·d^−1^ and the average daily intakes of rice for adult inhabitants were 0.221 kg·d^−1^ [33,34]; *C* is the metal concentration in the vegetable samples (mg·kg^−1^); *BW* = average body weight set to 70 kg for adults [35]; *AT* is averaging time for non-carcinogens: 365 × *EDd* (taken as 30 years for non-carcinogens and 70 years for carcinogens) [36].

The THQ Is calculated as follows:

(5) $$\mathrm{THQ}=\frac{EDI}{R_{f}D}$$

wherein, the *R_f_D* represents oral reference dose which for Cr (III) and Cr (VI) were 1.5 and 0.003 mg·kg^−1^·day^−1^, respectively.

If THQ is less than 1.0, there is no risk to human health, but if it is greater than 1.0, there is some degree of risk [36].

### 2.7. Carcinogenic Risk Assessment

Cancer risk (CR) was calculated according to the following equation:

(6) CR=EDI×CSF

where cancer risk represents the probability of individual lifetime health risks from carcinogens; *EDI* is the chronic daily intake of carcinogens (mg·kg^−1^·day^−1^); *SF* is the slope factor of hazardous substances (mg·kg^−1^·day^−1^). According to the Regional Screening Level (RSL) Summary Table [37], the *CSF* for Cr (VI) = 0.5 (mg·kg^−1^·day^−1^).

The permissible limits are within the range of 10^−6^–10^−4^ for a single carcinogenic element. In order to characterize the carcinogenic risk of Cr, the value of hexavalent Cr was used because only total Cr was analyzed. It is assumed that one-sixth of total Cr exists in the hexavalent form [38].

### 2.8. Statistical Methods

SPSS v22.0 (IBM Crop., Armonk, NY, USA) statistical software was used for data analysis, and statistical data were expressed as the average of three replicates with mean ± standard deviation (SD). One-way analysis of variance (ANOVA) and Duncan’s multiple comparison test was used with a significance threshold level of *p* < 0.05 level. OriginPro 2023 (Northampton, MA, USA) was used for data analysis and mapping. The relationships between variables were assessed by correlation analysis using principal component analysis (PCA) and Pearson’s correlation coefficients (*r*) for *p* < 0.05 using Originlab.

## 3. Results

### 3.1. Cr Content in Rhizosphere Soils of Different Crops

Cr pollution exists in different degrees in the surveyed area (Table 1). The Cr content in vegetable field C2 was up to 3883.77 mg·kg^−1^, which was 5.5, 7.8 and 18.13 times higher than that of rice field F1, and vegetable field C1 and C3, respectively. The Cr concentration of the investigated area was relatively high, among which Cr pollution of field C2 was the most serious and its Cr concentration was even higher than the risk intervention value of the National Soil Environmental Quality-Risk Control Standard for Soil Contamination of Agricultural Land (GB 15618-2018) in China. The Cr pollution index of vegetable field C3 was higher than 1 and less than 2, and that of paddy field F1 and most areas of vegetable field C1(except cabbage planting field) was 2–3. Therefore, these three fields were considered to have Cr moderate pollution. However, the Cr pollution index of the cabbage planting field in C1 exceeded 4, and that of C2 was higher than 25, so they were recognized as severe pollution.

### 3.2. Rhizosphere Soil Nutrient Contents in Different Crops

The data on rhizosphere soil pH, TOC, TN, AP, AK and CEC were significantly different (*p* < 0.05) (Table 2). In the three fields of F1, C1, and C2, except for the carrot, the pH of the soil where the other vegetables were planted was lower than 6.0, showing an acidic soil, and the pH of the soil where cabbage was planted was lower than 5, showing strong acidity. The content of TOC was relatively high, only that of lettuce rhizosphere soil was lower than 24 g·kg^−1^ (Table 2).

The TN of the soils which planted water spinach, edamame bean, and lettuce was lower than 3.0 g·kg^−1^, while others were higher than 3.0 g·kg^−1^(Table 2). The AP content was generally high, but the AP content of field C3 was significantly low and was one to two times lower than that of the other soils (*p* < 0.05). The content of AK in the soil for planting different vegetables was different. The AK in the taro rhizosphere was as high as 244.14 mg·kg^−1^ and that in the cabbage rhizosphere was as low as 94.64 mg·kg^−1^. The CEC content in rhizosphere soil was also relatively high, which in the taro rhizosphere was as high as 25.80 cmol^+^·kg^−1^ (Table 2).

### 3.3. Cr Concentration in Various Parts of Different Crops

Concentrations of Cr in different crop samples were determined (Table 3). The root Cr content of vegetable crops was significantly lower than that of rice (*p* < 0.05), in which the Cr content was 110.79 mg·kg^−1^. Among the vegetable crops, both the root and shoot Cr concentration in water spinach was the highest and significantly higher than that of the other vegetables (*p* < 0.05) (Table 2). Moreover, the content of Cr in the leaf or stem parts was in the range of 2.38–139.76 mg·kg^−1^, which in the seed was in the range of 2.18–2.85 mg·kg^−1^ (Table 3).

### 3.4. Cr Concentration and Enrichment Characteristics in Edible Parts of Different Crops

Compared with the national standard (GB2762-2017), the Cr content in the edible part of the majority of crops except for Chinese cabbage, taro and radish, exceeded the limitation level (Table 4). The Cr concentration in edamame bean and carrot exceeded the limitation value by about double, while that of water spinach was more than 60 times the standard level.

The transport capacity of crops was different, the TF of different crops’ edible parts occurred in the following order: Water spinach > Carrot > Radish > Mustard > Lettuce > Chinese cabbage > Cabbage > Rice > Taro (Table 4). Water spinach had strong transport ability, followed by the carrot and radish, but taro had poor transport ability to Cr.

Different crops have different bioaccumulation capacities in edible parts. The BCF of whole edible parts were Rice > Lettuce > Water spinach > Mustard > Carrot > Taro > Cabbage > Radish > Chinese cabbage > Edamame bean. Among them, taro had the strongest enrichment ability, with the BCF of edible parts reaching 1.85; the BCF of the edible parts of other crops were all less than 0.4.

The results of correlation analysis (Table 5) showed that there was a significant correlation between soil Cr content and Cr content in leaf or stem with a correlation coefficient of 0.964 (*p* = 0.01), but there was no significant correlation between Cr content in root and soil Cr content.

### 3.5. Potential Health Risk Assessment

The risk assessment was based on the concentrations of the individual metals in the edible parts of crops, the average consumption rate of crops and the body weight of the adult population. The EDIs based on these assumptions were not devoid of any error considering the fact that the toxicity of heavy metals to human health was proportional to their daily consumption [39]. The average EDI sequence of different crops was as follows: Water spinach > Rice > Edamame bean > Carrot > Lettuce > Chinese cabbage > Mustard > Taro > Cabbage > Radish. Among them, the EDI of water spinach was the highest (0.06 mg·kg^−1^·day^−1^), and the EDI of food crops was higher than the average EDI of leafy vegetable crops (except for water Spinach), root-tuber crops, and legume crop. Body weight and metal concentration are important factors to analyze THQ, and THQ varies greatly in different crops. The THQs of rice and cabbage were greater than 1.0, while the THQs of other crops were not greater than 1.0, indicating that the potential risk of rice and cabbage intake was relatively high, while the former risk of other crops was relatively low, and the present risk of white radish was the least (THQ < 0.1).

**Table 6:** The EDI and THQ of agricultural products.

Species		EDI (mg·kg^−1^·day^−1^)	THQ
Food crops	Rice	6.59 × 10^−3^	0.37
Leafy crops	Cabbage	1.92 × 10^−3^	0.11
	Chinese cabbage	2.62 × 10^−3^	0.15
	Water spinach	6.05 × 10^−2^	3.36
	Lettuce	3.71 × 10^−3^	0.21
	Mustard	2.55 × 10^−3^	0.14
Root-tuber crops	Carrot	4.44 × 10^−3^	0.25
	Taro	2.03 × 10^−3^	0.11
	Radish	1.46 × 10^−3^	0.08
Legume crops	Edamame bean	4.79 × 10^−3^	0.27

**Figure 1:**
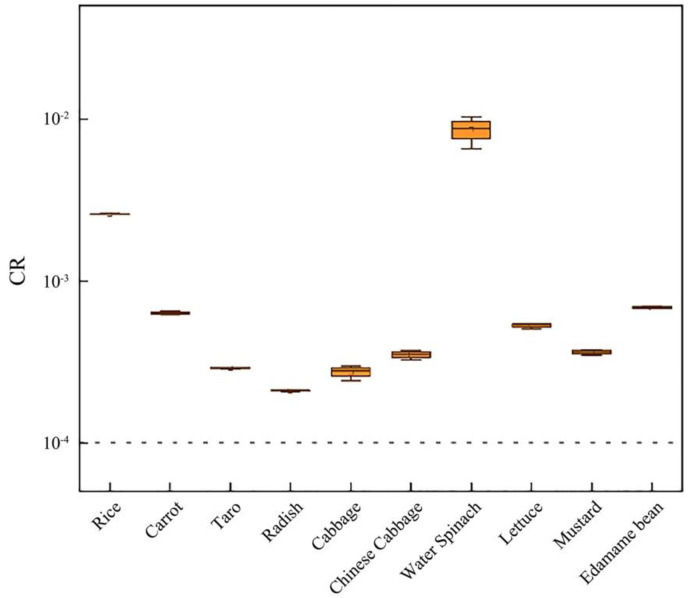
The carcinogenic risk (CR) of Cr associated with different crops. The dotted line indicates the limit value of high carcinogenic risk value.

In the farmland researched, the total carcinogenic risk of different farmland crops exceeded the acceptable limit of 10^−6^–10^−4^, so there was a serious carcinogenic risk for crops in this area. As shown in Figure 1, the carcinogenic risk of water spinach (CR = 8.58 × 10^−3^) and rice (CR = 2.58 × 10^−3^) was relatively high. In addition, the carcinogenic risk of edamame beans (CR = 6.84 × 10^−4^) and carrots (CR = 6.35 × 10^−4^) also existed to some extent. The carcinogenic risk of Cr in the edible part of crops followed the sequence of Water spinach > Rice > Edamame bean > Carrot > Lettuce > Mustard > Chinese cabbage > Taro > Cabbage > Radish. On the whole, the carcinogenic risk of different types of crops was shown as food crops > legume crops > leafy crops and root-tuber crops.

### 3.6. The Relationship between Soil Environmental Variables and Crops Indexes

**Figure 2:**
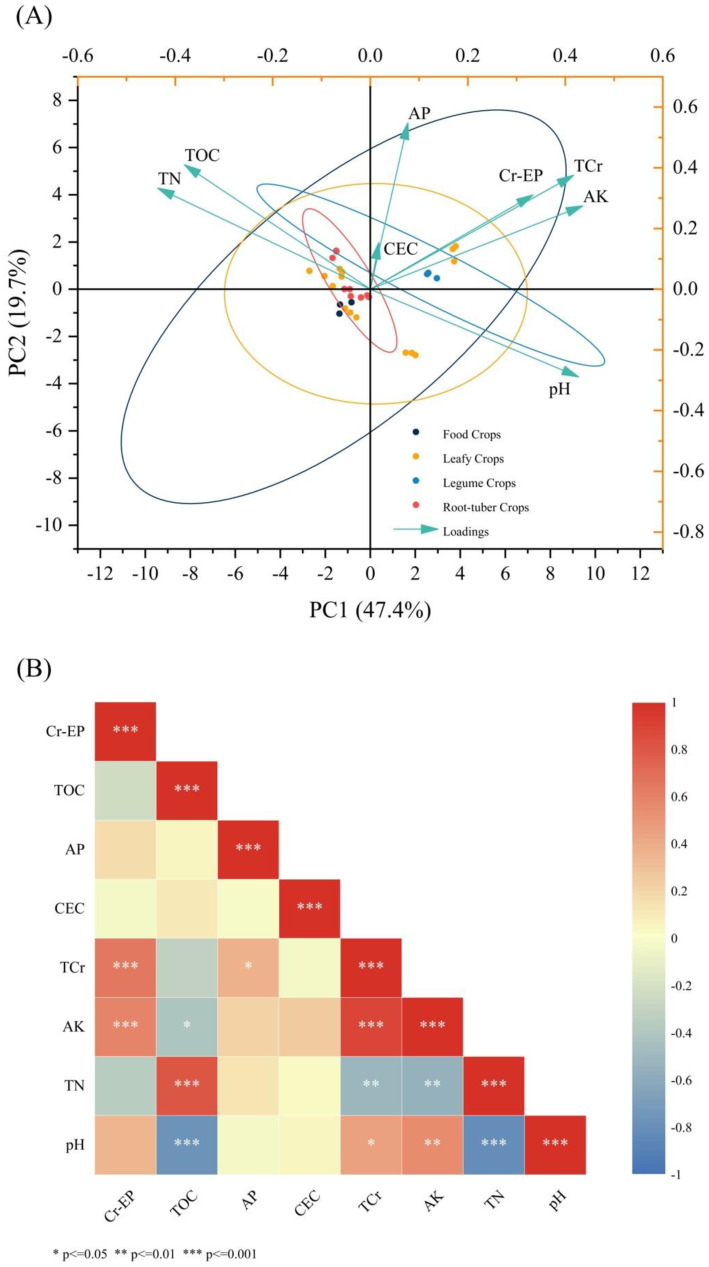
Principal component analysis (PCA) (**A**) and correlation analysis (**B**) for soil basic physicochemical properties, total Cr in soil, Cr bioaccumulation in the edible part of different crops. TCr: Total Cr in soil, Cr-EP: Cr in the edible part.

Using principal component analysis (PCA) and correlation analysis between soil environmental variables and Cr content in edible parts of crops, the important characteristics affecting Cr accumulation in edible parts of different crop types were revealed (Figure 2). The results showed that PC1 and PC2 explained 67.13% of the total variability in the data, which meant that soil environmental variables had a significant impact on the accumulation of Cr in edible parts of different types of crops.

The results of the Pearson correlation analysis among the Cr in the edible part were shown in Figure 2. The correlation coefficients among pH, TOC, TN, AP, AK, CEC, total Cr (TCr) in soil and Cr content in edible parts were significant at a *p* < 0.05 level. AK had a strong positive correlation with TCr in soil and Cr-EP (*p* < 0.05) and was directly related to Pearson phase 0.882 and 0.586 through the load. Additionally, there was a significantly positive correlation between total Cr in soil and Cr content in edible parts of plants (*r* = 0.659, *p* < 0.05). Other soil environmental variables had no significant relationship with Cr content in edible parts (*p* < 0.05); TOC (*r* = −0.227) and TN (*r* = −0.335) were negatively correlated with Cr in the edible part, while pH (*r* = 0.350) and AP (*r* = 0.162) were positively correlated with them, but there was no significant correlation (*p* < 0.05). In addition, pH (*r* = 0.459), TN (*r* = −0.501), and AP (*r* = 0.365) in the soil were significantly correlated with total Cr in the soil (*p* < 0.05).

## 4. Discussion

### 4.1. The Farmland Soil in the Area Was Contaminated with Cr

Compared with the screening value of the national standard (GB15618-2018) of China, the Cr content of the test farmland was generally higher, and there was relatively serious Cr pollution. Wang et al. reported the over-standard rate of Cr pollution in the Ningbo region was 60.48% [40]. In addition, it was also found in the relevant literature that the Cr content of farmland soil in the Ningbo region was 39.53 mg/kg [5], which was different from the experimental results, indicating the specificity of Cr pollution in this site. Additionally, the results showed that the Cr pollution levels of F1 (593.29 mg·kg^−1^), C1 (439.95 mg·kg^−1^), C2 (3888.77 mg·kg^−1^) and C3 (203.02 mg·kg^−1^) (Table 1) in the same area were different, which indicated that the Cr pollution in farmland had great spatial variability.

Through investigation and interview, it was found that this area had a long sewage irrigation history, which should be the main reason for the Cr pollution of this area [41]. Moreover, the farmland is located near a nearby national highway where the industries of aquaculture, plastic products, metal forging and others surrounded the farmland (Figure 1); 90% of total Cr ore production was used in the metallurgical industry for the production of steel, alloys and non-ferrous alloys [42]. So, production from the metallurgical industry might be one of the important sources of local Cr pollution. In addition, automobile exhaust emissions and tire wearing will produce a large number of harmful gases and dust containing heavy metal Cr, which will settle into the soil through atmospheric deposition, resulting in serious soil Cr pollution [43].

### 4.2. Crops Showed Different Enrichment and Transport Properties in Chrome-Contaminated Soils

Due to the spatial variability of Cr content in fields, the Cr content in different parts of the vegetables was different. However, by the correlation analysis, rhizosphere soil Cr content was significantly correlated with crop above-ground Cr content (Table 5) but had little correlation with underground Cr content. It was reported that there was a linear positive correlation between the Cr content of planting soil and the Cr content of leafy vegetable crops [44], and the trend was consistent with the results of this research.

The transport factor was used as an index to evaluate the heavy metal hyperaccumulation ability of plants. Cr accumulation in different plant tissues showed significant differences, with the highest content of Cr in the roots, which was consistent with the results of Edogb et al. [45]. The reason might be that most of the Cr in the soil was fixed in the vacuole of root cells after entering the plant body, which reduced the efficiency of Cr transport to the shoot [46]. It was also possible that after entering the root surface, Cr was reduced from Cr (VI) to Cr (III) when it crossed the endodermis through the symplast pathway so a great deal of Cr was retained in the root cortex cells [47,48]. The absorption of Cr (VI) by plants was an active process, and because Cr (VI) was structurally similar to sulfate and phosphate, it depended on the sulfate or phosphate carrier [49]. Among them, the transport coefficients of water spinach, radish, and carrot were higher than 1.0, which might be because the aboveground plants absorbed heavy metals from a non-soil environment through atmospheric deposition and other ways. Similar conclusions have been reported in the literature [50].

The bioconcentration factor was an index to evaluate the capacity of plants to absorb and transfer heavy metals into the body [30]. Cr had a lower migration rate than other metals such as Hg, Cd and arsenic (As) and primarily accumulated in plant roots [11]. The bioconcentration ability of different crops was different when they were planted in croplands polluted by Cr. In this study, the enrichment capacity of rice was greater than of leafy vegetables, rhizomes, and fresh legume crops, while the enrichment capacity of fresh legume crops was lower than that of leafy vegetables and rhizome crops. The bioconcentration characteristics of Cr in rice were more obvious than that in other agricultural products [51], which was similar to our results (Table 4). As the edible parts of root-tuber crops were mainly the roots or stems of crops, the edible parts of root-tuber crops were higher than other crops in theory because most of the chromium would be blocked in the roots when it entered the plant, the chromium content in the roots would be higher than that in the upper parts of the ground [46,47,48]. However, in the research, the Cr content of the edible parts of a few leafy vegetables was also higher than that of root-tuber crops, which might be related to the transport capacity of crops or the biomass of edible parts. Crops often showed the characteristics of short growth under chromium toxicity, so the Cr content of some edible parts might be too small, which increased the accumulation index [10]. In addition, some leafy vegetables and rhizome plants showed strong accumulation ability in several studies, and the accumulation of Cr in leafy vegetables was higher than that in fresh fruits and fruit vegetables grown under GCS [22,45]. Cr accumulation and distribution in plants varied among plant species and were influenced by the genetic and morphological characteristics of plants. In addition, various factors such as heavy metal concentration in soil, bioavailability, and soil physical and chemical properties would affect the absorption and enrichment of heavy metals in plants [40]. Due to the differences in physical and chemical properties of the soil of the collected crops, the differences in plant transport and accumulation capacity were also induced.

There was a certain correlation between soil physicochemical properties and Cr content in edible parts of crops. According to the correlation analysis results of PCA and Pearson (Figure 2), the soil environmental factors showed a certain correlation with the total Cr content of soil and the Cr content of plant edible parts. Among them, available potassium, Cr in soil, and Cr in edible parts showed a significant positive correlation (*p* < 0.05). This might be related to the effect of available potassium in soil on plant transport and absorption. Potassium had many important functions related to enzyme activation, as well as neutralization of negative charges, maintenance of cell expansion, and the increase in effective potassium for plant growth and organ movement [52], which might activate protein channels or ion channels for Cr absorption in plants, enabling plants to move from the ground to the ground and then to the edible parts. At present, the mechanism of Cr absorption and transport by plants in the soil has not been clearly and entirely studied. Recently, some researchers have demonstrated that sulfate transporters play a crucial role in the transport of Cr in roots [53]. What is more, it was reported that soil pH determined the chemical form of Cr in soil solution, and controlled the balance between solubility, adsorption and desorption of Cr in soil, and could even affect the surface charge, CEC and Eh of soil and other chemical and mineralogical properties to regulate the transport and redox behavior of Cr [54,55]. Moreover, organic matter also had a certain impact on the migration of soil Cr. Soil OM controls the bioavailability and morphology of Cr in soil mainly through three key mechanisms: adsorption, direct and indirect reduction [56]. However, in this study, the accumulation of plant edible parts was not only determined by the bioavailability of Cr in the soil but also related to the plant’s ability to enrich Cr, so the pH and OM in the soil might have no significant correlation with the content of Cr in the edible portion.

### 4.3. Crops Produced on the Cr Contaminated Farmland Have Health Risks

In agricultural activities, people will be exposed to toxic metals by ingestion, inhalation and dermal contact, of which the average daily intakes of metal ingestion were higher than that of the other two ways [39]. The average EDI sequence of water spinach and rice was relatively high (Table 6). The THQ of rice and cabbage was greater than 1.0, while that of other crops was basically not greater than 1.0. However, the carcinogenic risk of farmland crops exceeds the acceptable limit of 10^−6^–10^−4^ (Figure 1), so there was a serious carcinogenic risk for crops in this area. Although the nor-carcinogenic risk was not serious, the carcinogenic risk of each crop exceeded 10^−4^, indicating that local crops were not suitable for long-term consumption and need to be strictly controlled.

Therefore, according to the enrichment and transport properties of plants and the health risks of local crops, the heavily polluted farmland was not suitable for agricultural cultivation due to the spatial variability of soil Cr pollution. Although crops with low enrichment and transport capacities such as cabbage or taro could be planted safely in this area, the adjustment of cropping structure and remediation were urgently needed. Fortunately, based on this study and following the recent local policies, parts of the farmland in the area had been zoned for construction.

## 5. Conclusions

In this study, the quality and risk of Cr pollution in the local soil-crop system were evaluated by the single factor index method, THQ, CR and other evaluation methods, and combined with the national soil environmental quality standards and agricultural product quality and safety standards of China. We found that there was a serious Cr pollution problem in the surveyed farmland, and locally grown agricultural products were generally contaminated with Cr to a certain extent. Although the non-carcinogenic risk was generally not high, the carcinogenic risk exceeded the limit and there was a serious carcinogenic risk. Therefore, these results suggested that the health risks for locals exposed to farmland soil contaminated by Cr cannot be ignored and there were serious safety problems to be solved in the production of local agricultural products. The results were helpful to provide data support and guidance for chromium-polluted farmland safety production and remediation in the future.

## Figures and Tables

**Table 1:** Cr content and pollution index of *rhizosphere* soils.

Field	Species	Soil Cr Content (mg·kg^−1^)	Pi
F1	Rice	593.29 ± 209.54 b,c	2.37
C1	Carrot	400.89 ± 16.93 b–d	2.67
	Taro	381.39 ± 11.02 b–d	2.54
	Radish	338.05 ± 15.89 c,d	2.25
	Cabbage	414.04 ± 22.45 b–d	2.76
	Chinese cabbage	665.36 ± 63.80 b	4.44
C2	Water spinach	3883.77 ± 224.89 a	25.89
	Edamame bean	3883.77 ± 224.89 a	25.89
C3	Lettuce	223.27 ± 8.18 c,d	1.12
	Mustard	182.78 ± 11.62 d	1.22

Note: Data are average of three replicates ± SD. The different letters in the same column mean a significant difference at *p* < 0.05.

**Table 2:** Physiochemical properties of rhizosphere soils.

Field	Species	pH	TOC (g·kg^−1^)	TN (g·kg^−1^)	AP (mg·kg^−1^)	AK (mg·kg^−1^)	CEC (cmol^+^·kg^−1^)
F1	Rice	5.61 ± 0.05 d	39.70 ± 2.17 a,b	3.11 ± 0.40 b	157.43 ± 6.70 e	101.73 ± 29.97 d,e	15.79 ± 0.23 d
C1	Carrot	6.14 ± 0.04 c	31.40 ± 2.94 c	3.17 ± 0.33 b	157.43 ± 6.70 a,b	138.68 ± 12.52 c–e	16.03 ± 2.30 d
	Taro	5.31 ± 0.08 e	41.64 ± 1.99 a	4.08 ± 0.09 a	143.33 ± 1.67 b	244.14 ± 47.18 b	25.80 ± 0.59 a
	Ternip	5.30 ± 0.15 e	32.31 ± 1.20 c	3.15 ± 0.13 b	158.85 ± 3.94 a,b	110.88 ± 1.01 d,e	17.23 ± 0.32 c,d
	Cabbage	4.93 ± 0.11 f	40.57 ± 3.18 a,b	4.03 ± 0.35 a	98.82 ± 2.43 c	153.89 ± 21.46 c,d	16.63 ± 1.63 c,d
	Chinese cabbage	5.43 ± 0.07 e	37.26 ± 0.49 b	3.49 ± 0.20 a,b	177.74 ± 3.91 a	94.64 ± 12.02 e	19.54 ± 0.77 b,c
C2	Water spinach	6.40 ± 0.15 b	29.18 ± 0.46 c	2.22 ± 0.50 c	171.96 ± 3.77 a	443.29 ± 5.33 a	18.97 ± 0.74 b,c
	Edamame bean	6.40 ± 0.15 b	29.18 ± 0.46 c	2.22 ± 0.50 c	171.96 ± 3.77 a	443.29 ± 5.33 a	18.97 ± 0.74 b,c
C3	Lettuce	7.07 ± 0.09 a	23.27 ± 1.05 d	1.76 ± 0.12 c	48.02 ± 0.63 d,e	232.48 ± 32.54 b	20.62 ± 1.08 b
	Mustard	5.22 ± 0.06 e	30.66 ± 1.02 c	3.20 ± 0.40 b	55.35 ± 1.93 d	180.30 ± 4.26 c	18.51 ± 1.41 b–d

Note: Data are average of three replicates ± SD. The different letters in the same column represent a significant difference at *p* < 0.05.

**Table 3:** Cr content in different parts of crops and soil.

Field	Species	Cr Content in Plant Parts (mg·kg^−1^, DW)		
		Root	Leaf or Stem	Seed
F1	Rice	110.79 ± 25.54 a	7.55 ± 2.59 c	2.18 ± 0.42
C1	Carrot	6.03 ± 0.15 c	8.93 ± 1.16 c	n.k.
	Taro	39.64 ± 3.84 b	2.38 ± 0.26 c	n.k.
	Radish	8.19 ± 0.07 c	10.42 ± 0.84 c	n.k.
	Cabbage	44.51 ± 7.84 b	7.51 ± 0.81 c	n.k.
	Chinese cabbage	25.95 ± 1.48 b,c	9.63 ± 0.66 c	n.k.
C2	Water spinach	50.83 ± 4.89 b	139.76 ± 10.57 a	n.k.
	Edamame bean	n.k.	n.k.	2.85 ± 0.05
C3	Lettuce	47.45 ± 6.70 b	18.23 ± 1.21 b	n.k.
	Mustard	11.65 ± 0.89 c	7.99 ± 0.35 c	n.k.

Note: Data are average of three replicates ± SD. The different letters in the same column mean a significant difference at *p* < 0.05. n.k.: the data not known for their edible parts were not seed.

**Table 4:** Cr content and bioaccumulation coefficient of the edible part of agricultural products.

Species		TF	BCF (%)	Cr Content in EdiblePparts (mg·kg^−1^)	Quota of Cr (mg·kg^−1^)
Food crops	Rice	0.37	0.07	2.18 ± 0.42	1.0
Leafy crops	Cabbage	0.10	0.17	0.41 ± 0.01	0.5
	Chinese cabbage	0.08	0.37	0.55 ± 0.01	0.5
	Water spinach	0.33	2.75	12.80 ± 0.16	0.5
	Lettuce	0.35	0.38	0.78 ± 0.00	0.5
	Mustard	0.30	0.69	0.54 ± 0.01	0.5
Root-tuber crops	Carrot	0.23	1.48	0.94 ± 0.02	0.5
	Taro	0.11	0.06	0.43 ± 0.01	0.5
	Radish	0.09	1.27	0.31 ± 0.00	0.5
Legume crops	Edamame bean	0.03	n.k.	1.01 ± 0.02	1.0

Note: Quota of Cr refers to GB 2762-2017. The Cr content in the edible part of rice was calculated as dry weight, while the Cr content in the edible part of vegetable crops was calculated as fresh weight. Data are average of three replicates ± SD. n.k.: the data are not known.

**Table 5:** Pearson correlation analysis between each part of crops and soil Cr content.

Species	Root	Leaf or Stem	Soil
Root	1	0.145	0.207
Leaf or stem		1	0.976 **
Soil			1

Note: ** Correlation is significant at the 0.01 level (2-tailed).

## Data Availability

The data presented in this study are available on request from the corresponding author.

## Supplementary Materials

The following supporting information can be downloaded at: https://www.mdpi.com/2305-6304/11/1/27/s1, Figure S1: Sketch map of research area in Ningbo City. The areas outlined in the red line is the sampling site. The red stars represent some industries near the research area.
