# Supplementary material for: Chromium Contamination and Health Risk Assessment of Soil and Agricultural Products in a Rural Area in Southern China
Source: Toxics. 2022 Dec 27;11(1):27. doi: 10.3390/toxics11010027 (PMC9866242; doi:10.3390/toxics11010027)
Supplement: Supplementary file 1 [file toxics-11-00027-s001.zip › toxics-2109056-supplementary.pdf]

toxics

Supplementary Materials: Chromium Contamination and Health Risk Assessment of Soil and Agricultural Products in a Rural Area in Southern China

Shun'an Xu, Chao Yu, Qiong Wang, Jiaoyuan Liao, Chanjuan Liu, Lukuan Huang, Qizhen Liu, Zheyu Wen and Ying Feng

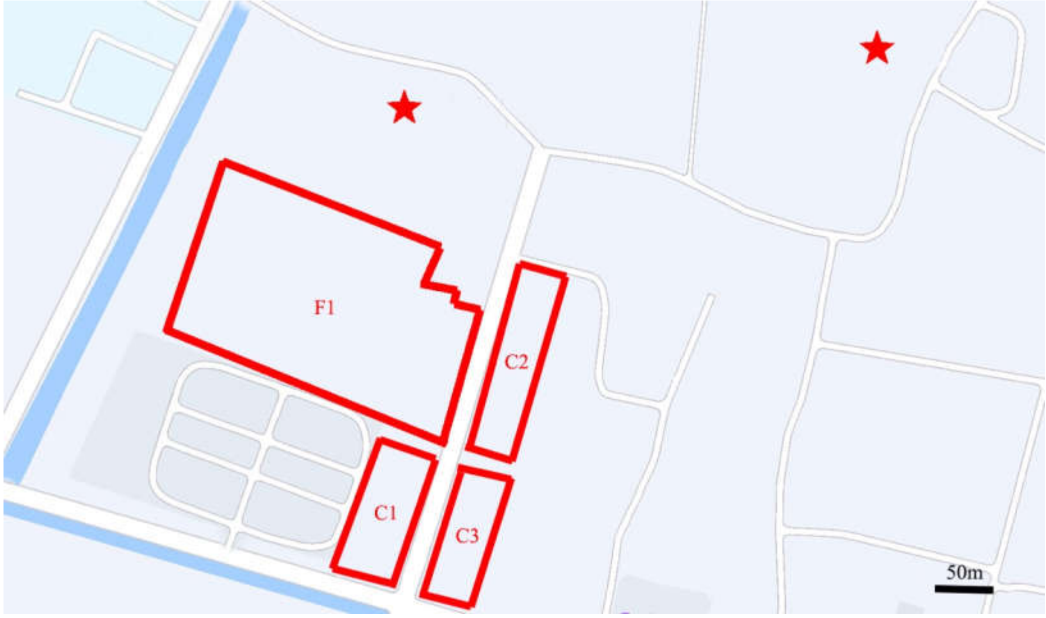

Figure S1. Sketch map of research area in Ningbo City. The areas outlined in the red line is the sampling site. The red stars represent some industries near the research area.
